# Association of vitamin D receptor gene polymorphism with type 1 diabetes mellitus risk in children: A protocol for systematic review and meta-analysis

**DOI:** 10.1097/MD.0000000000026637

**Published:** 2021-07-16

**Authors:** Yalin Ran, Suyuan Hu, Xiaohua Yu, Renjun Li

**Affiliations:** a Department of Pediatric, Chongqing Dadukou District People's Hospital, Chongqing, China; b Department of Pediatric, Chongqing Southeast Hospital, Chongqing, China.

**Keywords:** children, meta-analysis, polymorphism, protocol, type 1 diabetes mellitus, vitamin D receptorgene

## Abstract

**Background:**

Recent genetic association studies showed that there are contradictory results on the relationship between vitamin D receptor (VDR) gene polymorphisms and type 1 diabetes mellitus (T1DM) risk in children. The purpose of this systematic review is to collect the currently available evidence to evaluate the relationship between VDR gene polymorphisms and the risk of T1DM in children.

**Methods:**

Such medical databases as Wanfang Data, Chinese Biomedical Literature Database, Chinese National Knowledge Infrastructure, Chongqing VIP Chinese Science and Technology Periodical Database, PubMed, Embase, and Web of Science were extensively searched for relevant literatures published before June 2021 with the focus on the relationship between VDR gene polymorphisms and the risk of T1DM in children. The risk of bias was evaluated as per the Newcastle-Ottawa Scale by 2 independent researchers. Meta-analysis was performed to quantify the relationship between VDR gene polymorphisms and T1DM risk in children.

**Results:**

The results of this meta-analysis would be submitted to a peer-reviewed journal for publication.

**Conclusion:**

The relationship between VDR gene polymorphisms and T1DM risk in children is explored via this meta-analysis.

**Ethics and dissemination:**

Ethical approval was not required for this study. The systematic review will be published in a peer-reviewed journal, presented at conferences, and shared on social media platforms.

**Osf Registration Number:**

DOI 10.17605/OSF.IO/Q8XA5.

## 1 Introduction

Type 1 diabetes mellitus (T1DM) refers to an autoimmune disease that is mainly mediated by T cell immunity, and its pathogenesis is very complex.^[1–3]^ T1DM accounts for approximately 5% to 10% of all diabetes cases, and its prevalence is still on the rise.^[4]^ As is known to all, T1DM is a multi-factorial autoimmune disease induced by the interaction of genetic and environmental factors.^[5]^

In recent years, many genes related to T1DM have received extensive attention. It has been demonstrated in abundant studies that vitamin D may exert significant impacts on the pathogenesis of T1DM through vitamin D receptor (VDR) gene.^[6–8]^ Besides, it has been suggested in some studies that vitamin D deficiency is associated with the autoimmune destruction of β cells and the onset of T1DM induced by loss of immune regulation.^[9,10]^ Vitamin D has a protective effect on T1DM.^[11]^ Dietary vitamin D intake in early childhood could reduce the risk of T1DM.^[12,13]^ In addition, vitamin D supplementation during pregnancy may prevent the development of islet cell autoantibodies in newborns.^[14]^ Vitamin D could exert its actions via a nuclear vitamin D receptor (VDR).^[15]^ Therefore, the VDR gene can be considered a candidate/predisposing gene for T1DM.

In recent years, it has been demonstrated in related studies that VDR gene polymorphism may be associated with the genetic predisposition to T1DM. However, the relationship between VDR gene polymorphism and the risk of T1DM in children has not been confirmed yet, and there are contradictory results on such relationship.^[16–22]^ Therefore, the purpose of this study is to further verify the relationship between VDR gene polymorphism and the risk of T1DM in children by collecting relevant literature.

## 2 Methods

### 2.1 Study registration

The protocol of this review was registered in OSF (OSF registration number: DOI 10.17605/OSF.IO/Q8XA5). It was reported in accordance with the statement guidelines of preferred reporting items for systematic reviews and meta-analyzes protocol.^[23]^

### 2.2 Inclusion criteria

All eligible studies included in this study shall fulfill these inclusion criteria:

1. studies with the focus on the relationship between VDR gene polymorphisms and the risk of T1DM;
2. participants with the age less than 18 years old;
3. studies with sufficient data to calculate the odds ratio (OR) and its 95% confidence intervals (CIs);
4. case-control or cohort studies.

### 2.3 Exclusion criteria

The exclusion criteria were: letters, case reports, meta-analysis, review papers, papers without a control group, literature with abstract only, and literature without detailed genotype data.

### 2.4 Search strategy

Medical databases (Wanfang Data, Chinese Biomedical Literature Database, Chinese National Knowledge Infrastructure, Chongqing VIP Chinese Science and Technology Periodical Database, PubMed, Embase, and Web of Science) were systematically searched for papers published before June 2021 with respect to the relationship between VDR gene polymorphisms and the risk of T1DM in children. The search strategy for PubMed is presented in Table 1, and the corresponding keywords would be employed in other databases.

**Table 1:** Search strategy for PubMed.

Number	Search terms
#1	Diabetes Mellitus, Type 1[MeSH]
#2	Diabetes Mellitus, Brittle[Title/Abstract]
#3	Diabetes Mellitus, Insulin-Dependent[Title/Abstract]
#4	Diabetes Mellitus, Juvenile-Onset[Title/Abstract]
#5	Diabetes Mellitus, Ketosis-Prone[Title/Abstract]
#6	Diabetes Mellitus, Sudden-Onset[Title/Abstract]
#7	Diabetes, Autoimmune[Title/Abstract]
#8	IDDM[Title/Abstract]
#9	Autoimmune Diabetes[Title/Abstract]
#10	Diabetes Mellitus, Insulin-Dependent, 1[Title/Abstract]
#11	Diabetes Mellitus, Type I[Title/Abstract]
#12	Insulin-Dependent Diabetes Mellitus 1[Title/Abstract]
#13	Juvenile-Onset Diabetes[Title/Abstract]
#14	Type 1 Diabetes Mellitus[Title/Abstract]
#15	Brittle Diabetes Mellitus[Title/Abstract]
#16	Diabetes Mellitus, Insulin Dependent[Title/Abstract]
#17	Diabetes Mellitus, Juvenile Onset[Title/Abstract]
#18	Diabetes Mellitus, Ketosis Prone[Title/Abstract]
#19	Diabetes Mellitus, Sudden Onset[Title/Abstract]
#20	Diabetes, Juvenile-Onset[Title/Abstract]
#21	Insulin Dependent Diabetes Mellitus 1[Title/Abstract]
#22	Insulin-Dependent Diabetes Mellitus[Title/Abstract]
#23	Juvenile Onset Diabetes[Title/Abstract]
#24	Juvenile-Onset Diabetes Mellitus[Title/Abstract]
#25	Ketosis-Prone Diabetes Mellitus[Title/Abstract]
#26	Mellitus, Sudden-Onset Diabetes[Title/Abstract]
#27	Sudden-Onset Diabetes Mellitus[Title/Abstract]
#28	or/1-27
#29	Child[MeSH]
#30	Child∗[Title/Abstract]
#31	or/29-30
#32	Receptors, Calcitriol[MeSH]
#33	Calcitriol Receptors[Title/Abstract]
#34	Cholecalciferol Receptors[Title/Abstract]
#35	Receptors, Vitamin D[Title/Abstract]
#36	Vitamin D 3 Receptors[Title/Abstract]
#37	Vitamin D Receptors[Title/Abstract]
#38	1,25-Dihydroxycholecalciferol Receptor[Title/Abstract]
#39	1,25-Dihydroxycholecalciferol Receptors[Title/Abstract]
#40	1,25-Dihydroxyvitamin D 3 Receptor[Title/Abstract]
#41	1,25-Dihydroxyvitamin D3 Receptor[Title/Abstract]
#42	1,25-Dihydroxyvitamin D3 Receptors[Title/Abstract]
#43	Calcitriol Receptor[Title/Abstract]
#44	Receptors, 1,25-Dihydroxyvitamin D 3[Title/Abstract]
#45	Receptors, Cholecalciferol[Title/Abstract]
#46	Receptors, Vitamin D 3[Title/Abstract]
#47	Receptors, Vitamin D3[Title/Abstract]
#48	Vitamin D 3 Receptor[Title/Abstract]
#49	Vitamin D Receptor[Title/Abstract]
#50	Vitamin D3 Receptor[Title/Abstract]
#51	Vitamin D3 Receptors[Title/Abstract]
#52	1,25 Dihydroxycholecalciferol Receptor[Title/Abstract]
#53	1,25 Dihydroxycholecalciferol Receptors[Title/Abstract]
#54	1,25 Dihydroxyvitamin D 3 Receptor[Title/Abstract]
#55	1,25 Dihydroxyvitamin D3 Receptor[Title/Abstract]
#56	1,25 Dihydroxyvitamin D3 Receptors[Title/Abstract]
#57	D Receptor, Vitamin[Title/Abstract]
#58	D Receptors, Vitamin[Title/Abstract]
#59	D3 Receptor, 1,25-Dihydroxyvitamin[Title/Abstract]
#60	D3 Receptor, Vitamin[Title/Abstract]
#61	D3 Receptors, 1,25-Dihydroxyvitamin[Title/Abstract]
#62	D3 Receptors, Vitamin[Title/Abstract]
#63	Receptor, 1,25-Dihydroxycholecalciferol[Title/Abstract]
#64	Receptor, 1,25-Dihydroxyvitamin D3[Title/Abstract]
#65	Receptor, Calcitriol[Title/Abstract]
#66	Receptor, Vitamin D[Title/Abstract]
#67	Receptor, Vitamin D3[Title/Abstract]
#68	Receptors, 1,25-Dihydroxycholecalciferol[Title/Abstract]
#69	Receptors, 1,25-Dihydroxyvitamin D3[Title/Abstract]
#70	or/32–69
#71	Polymorph∗[Title/Abstract]
#72	Susceptibility[Title/Abstract]
#73	or/71–72
#74	#28 and #31 and #70 and #73

### 2.5 Data collection and analysis

#### 2.5.1 Selection of literature

**Figure 1:**
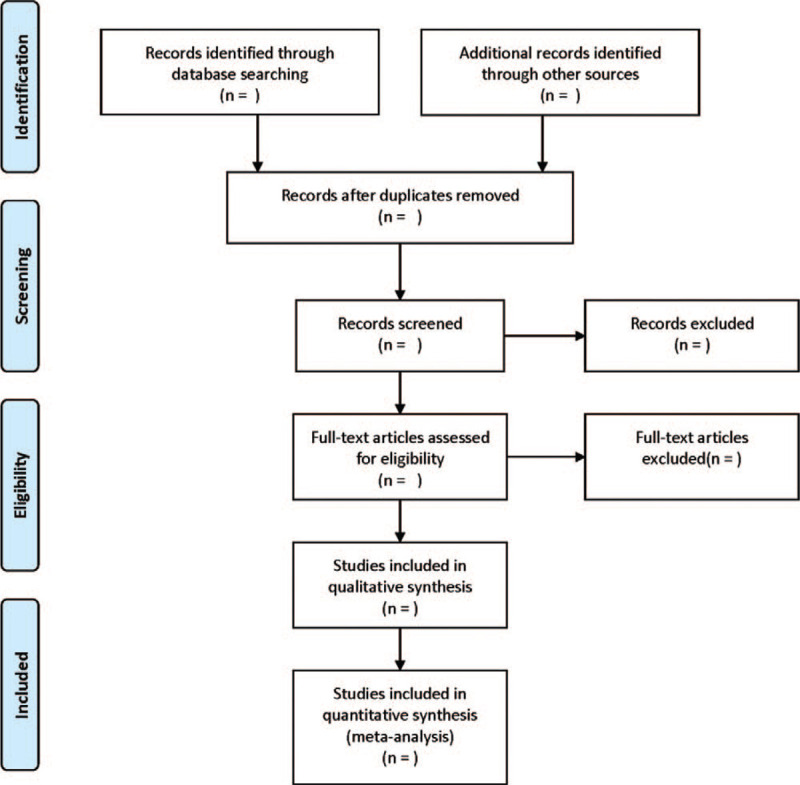
Flow chart of literature search and screen.

Two researchers independently screened the literature for data extraction and cross-checking. If there are differences, the discussion or consultation with a third party would be required. During the screening process, the title was read at first in an attempt to exclude the obviously irrelevant literature, followed by the abstract and the full text to determine its eligibility. The flowchart is presented in Figure 1.

#### 2.5.2 Data extraction

The following data were independently extracted by 2 researchers: first author, year of publication, country of origin, ethnicity, number of cases and controls, genotype frequency, source of controls, age, genotyping method, sample size, and Hardy-Weinberg equilibrium (HWE).

#### 2.5.3 Methodology quality assessment

The quality assessment of the included literature was investigated based on Newcastle-Ottawa Scale.^[24]^ Those with a score of 6 would be considered to be of high quality.^[25]^

#### 2.5.4 Dealing with missing data

In case of any missing data in a literature, please contact the newsletter author or the first author by email for accurate data. If there is a failure in the data request, descriptive analysis, instead of meta-analysis, shall be conducted.

#### 2.5.5 Statistical analysis

In each included literature, HWE was examined to assess bias in genotype distribution. Besides, odds ratios (ORs) and 95% confidence intervals (95% CIs) were calculated for analyzes of the VDR gene polymorphisms and T1DM risk in children. In addition, the pooled ORs and 95% CIs were calculated in 5 genetic models, namely allele model (T vs C), heterozygote model (TC vs CC), homozygote model (TT vs CC), dominant model (TT + TC vs CC), and recessive model (TT vs TC + CC). Moreover, the heterogeneity was calculated with the Chi-Squared-based *I*^2^ test and the Q test. If the *I*^2^ value is less than 50%, the fixed-effect model would be adopted. If the *I*^2^ value is more than 50%, a random-effects model would be adopted. All of the statistical analyzes were conducted by the STATA 16.0 (StataCorp, College Station, TX, USA), and the *P* values were two-sided.

#### 2.5.6 Subgroup analysis

According to ethnicity, source of controls and genotyping method, the subgroup analysis was performed on the relationship between VDR gene polymorphisms and the risk of T1DM in children.

#### 2.5.7 Sensitivity analysis

The eligible papers were sequentially removed in order to perform the sensitivity analysis.

#### 2.5.8 Assessment of publication biases

Potential publication bias was estimated by Egger linear regression test, and Begg test was employed to estimate the funnel plot asymmetry.^[26,27]^

#### 2.5.9 Ethics and dissemination

The content of this article does not involve moral approval or ethical review and would be presented in print or at relevant conferences.

## 3 Discussion

The VDR gene located at 12q13 consists of 9 exons and 8 introns, with multiple restriction endonuclease restriction sites. The common loci include BSM I, Apai, Taqi, and Foki.^[28]^ VDR gene polymorphisms have been associated with susceptibility to a variety of autoimmune diseases over the past few decades.^[29–31]^ In recent years, the relationship between VDR gene polymorphisms and T1DM has been investigated in several studies around the world. However, there is no meta-analysis of VDR polymorphism and the risk of T1DM in children. Meanwhile, the relationship between VDR gene polymorphisms and the risk of T1DM in children reported in the existing literature is inconsistent. In addition, the risk of T1DM is increasing due to vitamin D deficiency from year to year.^[32,33]^ Therefore, a comprehensive meta-analysis may be the optimal way to address these problems.

## Author contributions

**Conceptualization:** Renjun Li, Yalin Ran.

**Data curation:** Suyuan Hu.

**Funding acquisition:** Renjun Li.

**Formal analysis:** Xiaohua Yu.

**Investigation:** Suyuan Hu.

**Methodology:** Yalin Ran.

**Project administration:** Renjun Li.

**Resources:** Xiaohua Yu.

**Software:** Xiaohua Yu.

**Supervision:** Renjun Li.

**Validation:** Yalin Ran.

**Visualization and software:** Yalin Ran.

**Writing – original draft:** Yalin Ran and Renjun Li.

**Writing – review & editing:** Yalin Ran and Renjun Li.
